# Exposure of the mosquito vector *Culex pipiens* to the malaria parasite *Plasmodium relictum*: effect of infected blood intake on immune and antioxidant defences, fecundity and survival

**DOI:** 10.1186/s13071-016-1905-7

**Published:** 2016-11-29

**Authors:** Jessica Delhaye, Consolée Aletti, Olivier Glaizot, Philippe Christe

**Affiliations:** 1 Department of Ecology and Evolution, Biophore Unil Sorge, University of Lausanne, Lausanne, CH-1015 Switzerland; 2 Museum of Zoology, Place de la Riponne 6, Lausanne, CH-1005 Switzerland

**Keywords:** Exposure, Malaria, Trade-off, Vector

## Abstract

**Background:**

The intake of a *Plasmodium*-infected blood meal may affect mosquito physiology and a series of trade-offs may occur, in particular between immune defences, reproduction and self-maintenance. We evaluated the cost of exposure to *Plasmodium* in the mosquito vector by investigating the effect of exposure on fecundity and survival and the implication of immune and antioxidant defences in mediating this cost.

**Methods:**

We used the natural *Culex pipiens*-*Plasmodium relictum* association. We exposed female mosquitoes to increasing levels of parasites by allowing them to feed either on uninfected canaries, *Serinus canaria*, (unexposed mosquitoes) or on infected canaries with low (low exposure) or high (high exposure) parasitaemia. We recorded blood meal size, fecundity (laying probability and clutch size) and survival. We quantified the expression of genes involved in immune and antioxidant defences (nitric oxide synthase, NOS; superoxide dismutase, SOD; glucose-6-phosphate dehydrogenase, G6PDH).

**Results:**

We found that the laying probability of exposed females decreased with increasing exposure to the parasite and with increasing SOD expression. Clutch size of exposed females was higher compared to unexposed ones for similar blood meal size and was positively correlated to the NOS expression. We found no effect of exposure on survival. After blood meal intake, SOD increased in the three groups, NOS increased in exposed females and G6PDH increased in highly exposed females only.

**Conclusions:**

Our results illustrated a trade-off between fight against the parasite and reproduction and a cost of exposure which might be mediated by the investment in immune and/or antioxidant defences. They also showed that this trade-off could lead to opposed outcome, potentially depending on the vector physiological status. Finally, they highlighted that the ingestion of a *Plasmodium*-infected blood meal may affect mosquito life history traits in a complex way.

**Electronic supplementary material:**

The online version of this article (doi:10.1186/s13071-016-1905-7) contains supplementary material, which is available to authorized users.

## Background

Mosquito vectors are important protagonists in malaria epidemiology. They host the sexual reproduction of *Plasmodium* parasites responsible for malaria, leading to infectious sporozoite stages that reach the mosquito salivary glands, ready to be transmitted to a vertebrate host. *Plasmodium* infection is costly for mosquitoes, affecting (for instance) feeding behaviour [1, 2], resistance to nutritional stress [3], fecundity [4] and survival [5]. Yet, modifications of vector life history traits might occur due to the ingestion of a *Plasmodium*-infected blood meal only, even if the parasite does not develop.

*Plasmodium*-infected blood contains parasite stages as well as vertebrate host factors associated with infection that mosquitoes will be exposed to through their blood meal. Thus, the ingestion of a *Plasmodium*-infected blood meal may lead to physiological changes [6–12] that may be costly for the vector. First, exposure is followed by the activation of several immune effectors of the vector [13, 14] that may prevent *Plasmodium* development [15–20], such as unspecific and highly reactive pro-oxidant compounds. Pro-oxidants are a broadly used cytotoxic defence in invertebrates [21] affecting *Plasmodium* development [14, 22–24] but also harming the host which may suffer oxidative stress [25]. Mosquitoes may invest in antioxidant defences to protect themselves from oxidative damage [26, 27]. However parasites may also take advantage of the host antioxidant defences and a trade-off can occur in the mosquito between fight against the parasite and self-maintenance. For instance, it has been shown that mosquitoes experience local oxidative stress enhancement focused on the invasion site [26, 27] avoiding generalized oxidative damage [28]. Then, exposure is associated with vitellogenesis disruption, apoptosis and egg resorption in the ovaries [11, 29–32] that can lead to delayed reproduction and/or decreased fecundity [33]. This highlights a second trade-off between fight against the parasite and reproduction [34]. This trade-off has been suggested to originate from the amino acid (arginine) requirement needed for both immune response [21] and egg production [35]. It may also originate from the antioxidant requirement needed for both self-maintenance during the fight against the parasite and reproduction. Indeed, antioxidants are important in vector fecundity as exemplified by the involvement of catalase and superoxide dismutase in egg production [36, 37]. Therefore, following the intake of a *Plasmodium*-infected blood meal, mosquitoes should suffer costs from the exposure even if the parasite does not develop.

These studies have enabled a better understanding of the mechanisms and pathways involved in the mosquito-*Plasmodium* interaction. Taken all together, they allow us to predict that the investment in immune and antioxidant defences would mediate the cost of exposure and would lead to differential resource allocation into fight against the parasite, self-maintenance or reproduction. We proposed to investigate the effect of exposure to malaria parasite *Plasmodium relictum* (pSGS1) on fitness components of the mosquito vector *Culex pipiens*, a natural host-parasite association. We experimentally exposed female mosquitoes to *Plasmodium* parasites by allowing them to feed on experimentally infected canaries, *Serinus canaria*. It has been shown that the exposure level is related to the number of parasite blood stages ingested during the blood meal [38]. We tested three exposure levels - no, low and high exposure - by using three groups of birds, respectively: uninfected birds, birds in the chronic phase of infection carrying low parasite intensity (i.e*.* parasitaemia) and birds in the acute phase of infection with high parasitaemia. We measured two fitness components of the female mosquitoes: the fecundity measured as the oviposition occurrence as well as the clutch size after the first gonotrophic cycle and the survival, measured as the time from blood meal to death. We also investigated if the effect of *Plasmodium* exposure was linked with investment in immune and antioxidant defences. We sacrificed part of the females at 5 days post-feeding (dpf, corresponding to *Plasmodium* oocyst development in the midgut basal side) and at 15 dpf (corresponding to *Plasmodium* sporozoite invasion of the salivary glands) to quantify gene expression. We measured the expression of the immune gene nitric oxide synthase (NOS) producing the pro-oxidant molecule nitric oxide (NO), a broadly used cytotoxic defence in invertebrates [21]. We measured the expression of the antioxidant defence superoxide dismutase (SOD) detoxifying the pro-oxidant molecule superoxide (O_2_^−^), a pro-oxidant at the top of the pro-oxidative reaction chain. We also measured the expression of the glucose-6-phosphate dehydrogenase (G6PDH). It is an important enzyme implicated in the pentose phosphate pathway allowing the recycling of NADP^+^ to NADPH [39], which is involved in both antioxidant and immune pathways. In antioxidant pathways, NADPH can be used to produce the antioxidant glutathione and avoid oxidative damage. In immune pathways, it can be used by NOS to produce nitric oxide or by NADPH-oxidase (NOX) to produce superoxide pro-oxidants. This last mechanism, called respiratory burst, occurs in vertebrate macrophages and insect haemocytes during the defence against parasites [40, 41]. The pentose phosphate pathway is known to play a role in host-protozoa interaction [42], for instance a G6PDH mutation confers resistance to malaria in human populations [43]. We expected a trade-off between fight against the parasite and fecundity, with exposed females investing more in immune and antioxidant defences but laying less than unexposed females. We also expected the effect of exposure on investment in defences and on fecundity to increase with the exposure level.

## Methods

### Experimental design

#### Experimental infection of canaries

We used three different groups of birds to expose female mosquitoes to *Plasmodium* parasites. The first bird group was constituted with four uninfected canaries (uninfected group). Female mosquitoes feeding on these birds were not exposed to parasites and composed the control (unexposed) group. The second bird group consisted of four canaries experimentally infected *via* an intraperitoneal injection of 200 μl of blood from wild great tits (*Parus major*) naturally infected by *Plasmodium relictum* mixed with phosphate buffer saline (PBS, 1:1) two years before the beginning of the experiment. These canaries passed the acute phase of infection (peak of parasitaemia) and were in the chronic phase of infection [44], they had then low parasitaemia (chronic group). Mosquitoes feeding on them were then assumed to be exposed to a low amount of *Plasmodium* blood stages (low exposure). The third bird group consisted of four canaries experimentally infected *via* an intraperitoneal injection of 75 μl of a blood mix prepared from the four canaries of the chronic group mixed with PBS (1:1). In order to ensure a high parasitaemia in this last bird group, experimental infections were performed 10 days before the first mosquito feeding session to coincide with the acute phase of infection (acute group). Mosquitoes feeding on these birds were assumed to be exposed to a high amount of *Plasmodium* blood stages (high exposure).

We performed eight mosquito feeding sessions, using one canary of each bird group per feeding session, and using each bird twice. Prior to the first feeding session and once all the feeding sessions were performed, we blood sampled the birds in order to determine bird haematocrit (i.e. the fraction of red blood cells in the total blood volume) and bird parasitaemia in infected ones. Canaries from the acute group had significantly higher parasitaemia than canaries from the chronic group (Wilcoxon rank test: *W* = 0, *P* = 0.029; Additional file 1: Figure S1a). Mean haematocrit did not differ between the three bird groups (ANOVA: *F*_(2,9)_ = 1.08, *P* = 0.381, Additional file 1: Figure S1b).

#### Mosquito rearing and exposure to *Plasmodium*

We set up rainfall collecting containers (50 × 30 × 25 cm) in July 2014 at the forest of Dorigny (46°31′N, 6°34′E; alt. 380 m). Containers were initially filled up with water from Lake Geneva and baited with live yeast to favour visitation by gravid *Culex pipiens* females. From the 19th of August to the 10th of September, we collected *Culex pipiens* egg rafts in order to create independent clutch groups: eight groups of 25 ± 4 clutches (mean ± standard deviation). Clutches were allowed to hatch in the laboratory (24 °C, 65% relative humidity and a 12:12 h light–dark cycle) in individual containers filled up with 250 ml of mineral water. Larvae were then fed with commercial fish flakes (Tetra). The first emerging males of each clutch were morphologically determined to the species level and only clutches confirmed as *Culex pipiens* species were used. For each clutch group, seven days after the first emergence, adults were collected during 5 days, pooled in common cages (30 × 30 × 90 cm) and were provided with a 10% glucose solution. This allowed creating eight independent groups (each of them coming from one clutch group aforementioned and later called emergent group) of known age mosquitoes (age ± 2 days). Once an emergent group was created, females were kept 16 more days in cages with males for mating.

When females were 19 ± 2 days old, they were allowed to feed on one of the three different bird groups. For each emergent group, pools of 20 females were kept in cages (30 × 30 × 30 cm) 24 h before the feeding session and provided with water only. At the end of the 24 h, a first batch of three cages received one bird of each bird group (1 canary per cage). After 30 min, canaries were removed and placed in a second batch of three cages. This scenario was repeated to obtain as far as possible a total of 20 fed mosquitoes per bird over all the visited cages. Directly after each feeding session, fed females (presence of blood in the abdomen determined by eye) were placed separately in empty plastic vials (Sartsdet, 30 ml) provided with a 10% glucose solution during four days to collect haematin secretion for the estimation of the blood meal size. Haematin was stored at -80 °C until laboratory analyses and quantified *via* spectrophotometric measurement following previously described methods [45, 46]. Blood meal size was square root transformed to achieve normality.

At 5 days post-feeding (dpf), females were transferred into new vials containing 2.5 ml of water in order to allow them to oviposit and were fed with water only. Egg rafts were collected in order to measure clutch size as a proxy of fecundity. Females were then transferred into a third vial and provided with water only until death. We imposed a nutritional stress, a common stress factor in the wild, to the females because previous studies have shown that some effects of *Plasmodium* infection on vector life history traits might be stronger under stressful conditions [3]. All females were daily checked from the fifth dpf until death to record survival.

In order to quantify immune and antioxidant gene expression, ten females per exposure level and per feeding session were sacrificed at 5 and at 15 dpf (whenever possible five sacrificed females per dpf). To minimize variation between individuals, females were randomly chosen among females having already oviposited. Unexposed and exposed groups differed in the identity of the birds on which they fed, which can be an important confounding factor. We therefore compared these groups to a reference group. For this purpose, eight female mosquitoes of each emergent group were isolated before the feeding session and then sacrificed in order to assess the gene expression level prior to blood meal (reference baseline). Sacrificed and naturally dead females were stored at -80 °C until laboratory analyses. After mosquito dissection, wing size was measured as described in [47] and used as a proxy of body size.

We hypothesised that the complete development of the parasite in the vector might be associated with particular physiological changes in the mosquito vector (different intensities or different mechanisms compared to an aborted development). As we were interested by the cost of exposure when the parasite does not complete its life cycle, females which developed sporozoites in the salivary glands were excluded from the analyses.

### Laboratory measurements

In order to assess both infection status and investment in immune and antioxidant defences in a single individual, we dissected the mosquitoes and separated thorax and abdomen. This allowed us to extract DNA from the thoracic part for *Plasmodium* sporozoite detection in the salivary gland by PCR and to extract RNA from the abdominal part for gene expression quantification.

#### Plasmodium detection and quantification

Mosquito DNA was extracted from the thorax and bird DNA was extracted from the blood using the DNeasy blood and tissue extraction kit (Qiagen, Hilden, Germany), according to the manufacturer’s protocol. *Plasmodium* parasites were detected using PCR method previously developed by [48] and parasite quantification (in infected birds) was performed using quantitative real-time PCR as described in [49]. Parasitaemia was log-transformed to achieve normality.

#### Immune and antioxidant gene expression quantification

We measured the expression of one gene involved in mosquito immune response (nitric oxide synthase, NOS), one gene involved in antioxidant process (superoxide dismutase, SOD) and one gene involved in both immune and antioxidant pathways (glucose 6-phosphate dehydrogenase, G6PDH). Total RNA was extracted from the mosquito abdomen using Trizol reagent (Life Technologies, Invitrogen, Carlsbad, California, USA) and treated with Deoxyribonuclease I, Amplification Grade (DNase I, Amp Grade, Invitrogen, Carlsbad, California, USA), according to the manufacturers’ protocols. Total RNA was quantified using NanoDrop spectrophotometer (Thermo Fisher Scientific, Waltham, Massachusetts, USA). Retrotranscription of RNA to cDNA was performed from 50 ng of total RNA using PrimeScript RT reagent kit (Takara Bio, Kusatsu, Japan), following the manufacturer’s protocol. The qPCR reaction was performed using PowerUp SYBR Green Master Mix (Thermo Fisher Scientific) according to the manufacturer’s protocol and cDNA samples diluted to 1:10. The primer sequences used were: NOS, 5′-CGA GAA GGC CCA CAT CTA CG-3′ and 5′-CGA CAG CAT GTA CTT CTC CA-3′; SOD, 5′-GCA TTG CGA AAA CTT CCT TC-3′ and 5′-TGC CCA GAT CAT CAA TTT CA-3′; G6PDH, 5′-CGC GCA CGA GGA AAA GTA CG-3′ and 5′-GGT TTG CGG TCT TCC CAA CC-3′ and rpl19, 5′-CGC TTT GTT TGA TCG TGT GT-3′ and 5′-CCA ATC CAG GAG TGC TTT TG-3′ as reference gene [36, 50, 51]. The qPCRs were run in duplicates with an AB 7500 qPCR system (Applied Biosystems, Foster City, California, USA) as follows: 2 min at 50 °C, 10 min at 95 °C followed by 40 cycles: 15 s at 95 °C, 1 min at 60 °C. A melting curve was produced at the end of each run to control for amplification specificity. A standard curve was produced for each gene with 2-fold serial dilutions of cDNA. Expression of each gene of interest was calculated the same way that bird parasitaemia and as described in [49]. Briefly, for each measured gene, DNA concentration was calculated from a standard curve and the gene expression level was given by the ratio of the DNA concentration of the gene of interest on the DNA concentration of the reference gene. Expression level of each gene was log-transformed to achieve normality. One female, which was sacrificed at 5 dpf and which received high exposure, was discarded from the analyses because of particularly high gene expression values (outsider).

### Statistical analyses

Statistical analyses were performed with R (version 3.1, [52]). We had two measurements per bird for parasitaemia (before the first feeding session and at the end of all the feeding sessions), then we calculated the mean values for each individual and used mean bird parasitaemia in the analyses.

We analysed blood meal size as a response variable in a linear mixed effects model (lmer function in *lme4* package) including terms for body size, exposure level (unexposed - low exposure - high exposure) and the interaction between both. For the fecundity, we first analysed the laying probability as a response variable in a generalized mixed effects model (glmer function in lme4 package) including terms for blood meal size, exposure level and the interaction between both. Considering mosquitoes which oviposited only, we also analysed clutch size as a response variable in a lmer model including terms for body size, blood meal size, exposure level and the interactions between body size and exposure level and between blood meal size and exposure level. Considering all females which died naturally, we analysed survival as a response variable in mixed effects cox model (coxme function in *coxme* package) including terms for blood meal size, fecundity, exposure level and the interactions between blood meal size and exposure level and between fecundity and exposure level. For all models, we included terms for emergent group and canary identity as random factors. We also ran the models considering low and high exposure levels only, replacing exposure level by bird parasitaemia and removing canary identity from the random factors to investigate the effect of exposure level as a quantitative continuous variable. The detailed model structures are described in Additional file 2: Table S1.

For each gene measured, we first compared the gene expression level of each group of fed females (5 no - 5 low - 5 high - 15 no - 15 low - 15 high, dpf exposure) to the gene expression level of sacrificed unfed females with a one-way Anova and emergent group as a random factor (lme function in nlme package). Finally, in order to look at the effect of gene expression on fecundity, we considered exposed females sacrificed at 15 dpf and analysed laying probability and clutch size as response variables and included terms for expression levels of each gene and exposure level as well as the two-way interactions between each gene expression level and exposure level in lmer and glmer models (for clutch size and laying probability respectively) and considering emergent group and canary identity as random factors. The detailed model structures are described in Additional file 2: Table S1.

For each model, we performed backward model selection based on likelihood ratio tests until reaching the minimal adequate model [53]. After model selection, we performed contrast analyses to look at the effect of each term individually. The significant *P*-values given in the text come from the minimal adequate models and the non-significant *P*-values come from the likelihood ratio tests prior to the elimination of the non-significant term from the model.

## Results

### Mosquito exposure

At the end of all the feeding sessions, 156 female mosquitoes took a blood meal on uninfected canaries, 199 took a blood meal on canaries from the chronic group and 162 took a blood meal on canaries from the acute group. Details about sample sizes are given in Table 1.

**Table 1:** Sample sizes of female (♀) mosquitoes obtained at the end of all the feeding sessions

♀ Fed on:		Exposure level	Total	Died naturally	Sacrificed	
Bird group	Bird parasitaemia				At 5 dpf	At 15 dpf
Uninfected	none	no exposure	156	85	36	35
Chronic	low	low exposure	199	119	40	40
Acute	high	high exposure	162	83	40	39
♀ which did not take a blood meal		no exposure	64	0	64	

Sample sizes are given for females fed on the different bird groups (with corresponding parasiteamia and exposure level) and for unfed females. For each exposure level, the total sample size, as well as the sample size of females which died naturally and females which were sacrificed at 5 and at 15 days post-feeding (dpf, 0 dpf for unfed females) are given

### Blood meal size, fecundity and survival

Blood meal size increased with mosquito body size (Table 2). In exposed females, blood meal size slightly increased with bird parasitaemia (Table 2).

**Table 2:** Minimal adequate models for blood meal size, fecundity and survival

Categorical exposure level: no – low – high exposure						Quantitative continuous exposure level: bird parasitaemia					
Lmer		Estimate	SE	*t*-value	*P*	Lmer		Estimate	SE	*t*-value	*P*
Blood meal size (square-root transformed)						Blood meal size (square-root transformed)					
	Intercept	0.17	0.87	0.20	0.845		Intercept	0.74	0.99	0.75	0.455
	Body size	1.13	0.25	4.56	< 0.001		Body size	0.95	0.29	3.32	0.001
							Bird parasitaemia	0.14	0.06	2.40	0.016
R^2^_m_ = 3.4%; R^2^_c_ = 19.1%						R^2^_m_ = 4.5%; R^2^_c_ = 10.8%					
Clutch size						Clutch size					
	Intercept	-146.28	48.90	-2.99	0.003		Intercept	-147.60	54.87	-2.69	0.008
	Body size	74.58	14.58	5.24	< 0.001		Body size	66.28	16.15	4.10	< 0.001
	Blood meal size	2.76	0.43	6.36	< 0.001		Blood meal size	4.81	0.37	12.97	< 0.001
	Exposure level	-30.08	16.65	-1.82	0.076						
	Blood meal : exposure	2.15	0.72	2.95	0.006						
R^2^_m_ = 48.5%; R^2^_c_ = 50.6%						R^2^_m_ = 50.9%; R^2^_c_ = 52.5%					
Coxme		Exp(coef.)	SE	*z*-value	*P*	Coxme		Exp(coef.)	SE	*z*-value	*P*
Survival						Survival					
	Clutch size	1.01	0.001	10.29	< 0.001		Clutch size	1.01	0.001	9.83	< 0.001
Glmer		Estimate	SE	*z*-value	*P*	Glmer		Estimate	SE	*z*-value	*P*
Laying probability						Laying probability					
	Intercept	-0.57	0.35	-1.60	0.110		Intercept	-0.56	0.33	-1.67	0.094
	Blood meal size	0.09	0.02	4.82	< 0.001		Blood meal size	0.09	0.02	4.21	< 0.001
	Exposure level				0.064		Bird parasitaemia	-0.45	0.16	-2.86	0.004
R^2^_m_ = 18.9%; R^2^_c_ = 26.4%						R^2^_m_ = 18.2%; R^2^_c_ = 18.2%					

Each response variable (blood meal size, clutch size, survival and laying probability) was analysed with exposure level as a categorical variable (unexposed – low exposure – high exposure) and as a quantitative continuous variable (bird parasitaemia). Minimal adequate models, obtained after backward model selection based on likelihood ratio tests, are given with estimate or coefficient (exponential value, Exp(coef.)), standard error (SE), *t*- or *z*-value and *P*-value associated with each parameter in accordance with the performed models (lmer, coxme or glmer). Marginal and conditional R^2^ (R^2^_m_ and R^2^_c_, respectively) were calculated for mixed effects models according to [63]

**Fig. 1:**
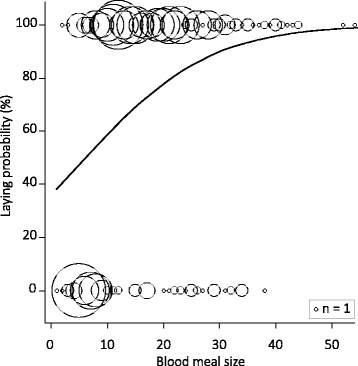
Laying probability as a function of blood meal size. Fecundity measured as laying probability (in percent) of females that died naturally as a function of blood meal size from the minimal adequate model given in Table 2. Circle size represents mosquito sample size (*n*)

**Fig. 2:**
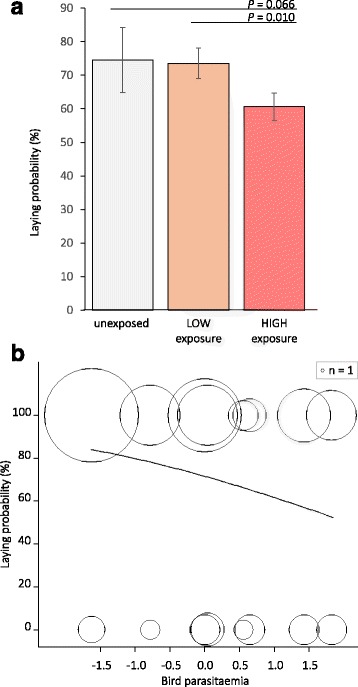
Laying probability as a function of exposure to *Plasmodium* parasites. Fecundity measured as laying probability (in percent) as a function of **a** exposure level in females that died naturally, and **b** bird parasitaemia (circle size represents mosquito sample size) in exposed females that died naturally from the minimal adequate model given in Table 2

Laying probability increased with blood meal size (Table 2, Fig. 1) and tended to decrease with increasing exposure level (*P* = 0.064): females under high exposure had lower probability to lay than females under low exposure (*P* = 0.010) but there was a marginally significant difference between females under high exposure and unexposed females due to higher variation in the unexposed group (*P* = 0.066, Table 2, Fig. 2a). The effect of exposure level on laying probability was clearer when considering exposure level as a quantitative continuous variable and in exposed females, laying probability decreased with bird parasitaemia (Table 2, Fig. 2b).

**Fig. 3:**
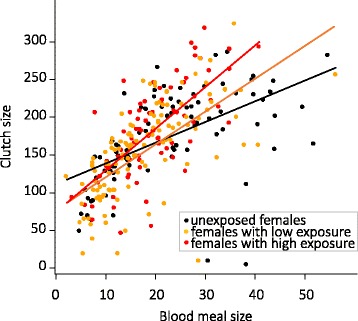
Clutch size as a function of blood meal size and exposure level. Fecundity measured as clutch size in females that oviposited as a function of blood meal size and exposure level from the minimal adequate model given in Table 2

Clutch size increased with body size (Table 2). There was also a significant interaction between blood meal size and exposure level to *Plasmodium* (Table 2): clutch size increased with blood meal size and for similar blood meal size exposed females laid more eggs compared to unexposed ones (Table 2, Fig. 3). This difference of blood-to-egg conversion increased with blood meal size (Fig. 3). There was no effect of bird parasitaemia on clutch size (Table 2).

Only clutch size significantly explained survival and females laying more eggs died faster (Table 2).

### Gene expression

**Fig. 4:**
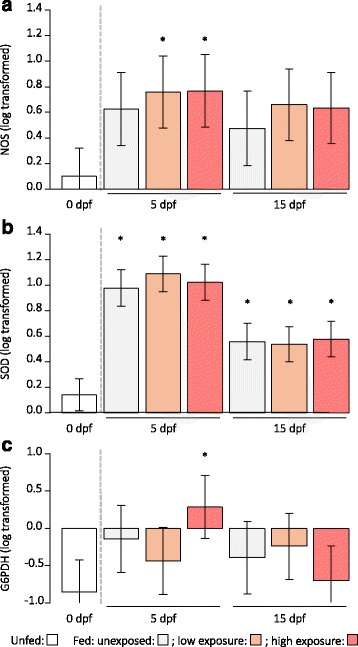
Immune and antioxidant defences as a function of exposure level and days post- feeding. Gene expression levels of **a** nitric oxide synthase (NOS, log-transformed), **b** superoxide dismutase (SOD, log-transformed) and **c** glucose 6-phosphate dehydrogenase (G6PDH, log-transformed) in unfed female mosquitoes and in fed female mosquitoes: unexposed, with low exposure and with high exposure to *Plasmodium* parasites at 5 and 15 days post-feeding (dpf). Stars indicate significant statistical differences compared to unfed females at 0 dpf

Compared to unfed female mosquitoes, NOS level was significantly higher in exposed females at 5 dpf (low exposure: *P* = 0.021, high exposure: *P* = 0.027, Fig. 4a). NOS levels in the other groups were not significantly different from unfed females. All fed females had increased SOD level with a 10-fold increase at 5 dpf (no – low – high exposure at 5 dpf: all *P* < 0.001) and a 5-fold increase at 15 dpf (no - low exposure at 15 dpf: both *P* = 0.002, high exposure at 15 dpf: *P* < 0.001) compared to unfed females (Fig. 4b). Compared to unfed females, only highly exposed females at 5 dpf had a higher G6PDH level (Fig. 4c). None of the gene expression levels were correlated with body size, blood meal size or bird parasitaemia (in exposed mosquitoes), but SOD level was increasing with NOS and with G6PDH levels (Table 3).

**Table 3:** Pearson correlations

Gene	Correlation with	cor	*n*	*P*
NOS	SOD	**0.30**	**286**	**< 0.001**
	G6PDH	0.10	277	0.240
	Body size	0.10	286	0.240
	Blood meal size	0.04	223	0.650
	Bird parasitaemia	0.09	155	0.321
SOD	G6PDH	**0.23**	**280**	**< 0.001**
	Body size	0.08	289	0.321
	Blood meal size	0.12	226	0.240
	Bird parasitaemia	0.09	156	0.394
G6PDH	Body size	-0.07	280	0.321
	Blood meal size	0.09	219	0.321
	Bird parasitaemia	0.01	152	0.915

For each gene expression level (log-transformed): nitric oxide synthase (NOS), superoxide dismutase (SOD) and glucose-6-phosphate dehydrogenase (G6PDH), the correlation with the other genes, mosquito body size, blood meal size and bird parasitaemia (in exposed female mosquitoes) was tested. Correlation coefficient (cor), sample size (*n*), and *P*-value (adjusted for multiple comparisons) are given. Significant correlations are indicated in bold

**Fig. 5:**
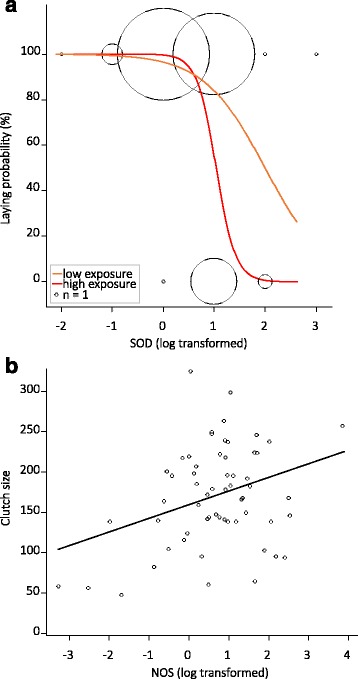
Fecundity and gene expression in exposed females sacrificed at 15 days post-feeding. **a** Laying probability as a function of superoxide dismutase level (SOD, log-transformed) and exposure level from the minimal adequate model given in Table 4. Circle size represents mosquito sample size. **b** Clutch size as a function of nitric oxide synthase level (NOS, log-transformed) from the minimal adequate model given in Table 4

In exposed females sacrificed at 15 dpf, there was a marginal effect of the interaction between SOD and exposure level on laying probability. Laying probability decreased with SOD level and the decrease was more important in highly exposed females compared to females receiving a low exposure (Table 4, Fig. 5a). There was no effect of NOS or G6PDH levels (Table 4). Among females that laid eggs, clutch size increased with NOS level (Table 4, Fig. 5b) but there was no effect of SOD and G6PDH expressions or exposure level (Table 4).

**Table 4:** Minimal adequate models for fecundity as a function of gene expression

Lmer	Est.	SE	t-value	P
Clutch size				
Intercept	160.53	9.09	17.67	< 0.001
NOS level	16.62	6.21	2.68	< 0.001
R^2^_m_ = 11.3%; R^2^_c_ = 13.1%				
Glmer				
Laying probability				
Intercept	5.47	1.84	2.97	0.003
SOD level	-5.28	1.78	-2.96	0.003
Exposure level	-2.17	1.98	-1.09	0.275
SOD : exposure	3.62	1.99	1.81	0.069
R^2^_m_ = 58.8%; R^2^_c_ = 71.0%				

For each response variable, clutch size and laying probability, minimal adequate models, obtained after backward model selection based on likelihood ratio tests, are given with estimate (Est.), standard error (SE), *t*- or *z*-value and *P*-value associated with each parameter in accordance with the performed models (lmer or glmer). Marginal and conditional R^2^ (R^2^_m_ and R^2^_c_, respectively) were calculated according to [63]

## Discussion

In this study, we investigated the effect of exposure to *Plasmodium* parasites on the mosquito vector *Culex pipiens* fecundity, survival and investment in immune and antioxidant defences. Firstly, we found a cost of exposure to *Plasmodium* parasites on the vector fecundity as laying probability decreased with increasing exposure level (measured as bird parasitaemia). Laying probability also decreased with increasing SOD expression in exposed females. This effect suggested a trade-off between fight against the parasite/self-maintenance and reproduction. Secondly, among females that laid eggs, exposed females had a higher blood-to-egg conversion compared to unexposed ones and their clutch size also increased with NOS expression. These results depicted a link between investment in immune defences and fecundity. Thirdly, only highly exposed females showed a higher expression of G6PDH, suggesting the involvement of that gene during a vector-*Plasmodium* interaction. Finally, we found no effect of exposure to *Plasmodium* on the vector survival.

*Plasmodium* parasites are known to affect vector fecundity with, for instance, egg resorption occurring in the mosquito ovaries during *Plasmodium* development [11, 29–32]. Here, we found that laying probability increased with blood meal size: a particularly small blood meal being not sufficient to mature eggs [54]. This taken into account, we found that exposure to *Plasmodium* imposed a cost on the vector fecundity as laying probability decreased with increasing exposure to *Plasmodium* (measured as bird parasitaemia) as well as with increasing SOD expression. All fed females, regardless of their exposure level, expressed similarly SOD showing no specific investment in this antioxidant defence after *Plasmodium* exposure but simply a protective role after blood intake [55]. All exposed females, regardless of the low or high exposure, expressed more NOS. Although uninfected blood ingestion may trigger immune activation in the vector due to the presence of immunogenic factors in the vertebrate blood, a stronger activation as well as the activation of specific genes after a *Plasmodium*-infected blood meal have been described [6–10]. Our results suggested a higher oxidative stress in exposed females compared to unexposed ones (i.e*.* same amount of antioxidant, but more pro-oxidants in exposed females compared to unexposed ones). Finally, only highly exposed females were expressing more G6PDH at 5 dpf. It is the first time that this gene is shown to be involved during a vector-*Plasmodium* interaction. This result suggested that the activation of some physiological pathways might depend on the exposure level and occur above a certain threshold of exposure. G6PDH is an enzyme involved in the pentose phosphate pathway allowing the transformation of NADP^+^ in NADPH [39]. It is involved in both immune and antioxidant pathways through its implication in NO, O_2_^−^ and glutathione production. Our data do not allow to discriminate between these processes. Yet, the laying probability of exposed females decreased with an increasing exposure level, an increasing SOD level and with the expression of G6PDH. All together, these results corroborate a trade-off between fight against parasites/self-maintenance and reproduction associated with an investment in immune and/or antioxidant defences that may mediate the trade-off.

Previous studies [11, 29–32] have been looking at mosquito fecundity *via* dissection of the ovaries without allowing females to oviposit. Some studies measured fecundity as the clutch size after oviposition and observed a decreased clutch size with infected blood meal intake [33]. Here, exposed females that oviposited had a higher blood-to-egg conversion compared to unexposed ones. A positive effect of *Plasmodium* exposure on the vector fecundity has already been reported in [56] where the prevalence of gravid females (females producing eggs in the ovaries) was higher in the group taking an infected blood meal than in the group taking an uninfected blood meal. If this result would be due to different blood quality (e.g*.* amount of resources) between non-infected and infected blood, we could expect a fixed conversion difference between unexposed and exposed females. This was not the case here as the conversion difference increased with blood meal size. Alternatively, it has also been shown in *Culex pipiens* that parasitism influences reproductive life-history traits with for instance delayed hatching date of eggs laid after a *Plasmodium*-infected blood meal [33] or larvae pupating faster and reproducing earlier to compensate for decreased survival when infected by microsporidian parasites [57]. In our case, a higher blood-to-egg conversion might either compensate a potential impaired hatching success or, for similar hatching rate, enhance reproductive success in exposed females compared to unexposed ones. This could be part of a fight against parasites-reproduction trade-off. Indeed, the first gonotrophic cycle is important for female mosquitoes, because several risks increase with subsequent reproduction events: risk of death at each blood meal intake [58–60], decreased fertility with age [37] and with infection [29, 61]. Therefore, maximizing the first reproduction when taking an infected blood meal may be beneficial. Finally, the positive link observed between clutch size and NOS expression could either reflect the condition status of the females: the better the female, the more eggs and the higher the immune response, or suggests that investing in immune defences could act as a signal for investing in egg maturation in a proportional way. Even though our data do not provide a terminal measurement of reproductive success (e.g*.* hatching rate) of the females and do not allow us to conclude about the underlying mechanism, these results showed a positive link between investment in immune defences and fecundity (measured as clutch size). This positive link contrasts with the former result showing a decreased fecundity (measured as laying probability) with an increased exposure to *Plasmodium*. This dual response could depend on physiological signals perceived by individuals. For instance, the amount of ingested parasites, the strength of a particular gene activation (or downregulation) or the engagement in a particular physiological pathway (e.g*.* G6PDH more expressed in highly exposed females only) may modify the vector physiological status. Such modifications could act as cues and contribute to determine if females will invest in self-maintenance or in reproduction. We could thus expect that the stronger the cues the female is receiving, the higher the investment in reproduction and the higher the probability of developing *Plasmodium* infection reaching the salivary glands. However, several factors may affect the probability of infection and lead to very contrasting results. For instance, if infection depends on individual quality, we would expect infected individuals to present a lower investment in immune and antioxidant defences and a lower reproductive success. In contrast, if infection depends on the number of ingested blood parasites only, we would expect no relationship between life history traits and infection. Supplementary data on *Plasmodium*-infected female mosquitoes could help understanding the involved underlying mechanisms.

While some of our present findings were in the direction of the predictions and supported previous studies, others were counterintuitive. The effect of *Plasmodium* parasites on vector life history traits will depend on intrinsic characteristics of the host-parasite association under study. Such characteristics are the interspecific interaction (what are the interacting species?), the nature of the association (i.e*.* natural *versus* artificial) and the time since the (host and parasite) populations have been isolated from the wild. Indeed, naturally occurring associations share an evolutionary history that has led the interacting species to express specific exploitation and defence strategies. These strategies will depend on the interacting species and might be absent in artificial associations. Furthermore, the isolation of individuals from the wild to controlled laboratory conditions for several generations alters the selective pressures imposed on them and may modify the expression of wild traits. In research conducted on vector-*Plasmodium*, studies may differ substantially in terms of these characteristics [62]. Here, we studied a natural (*Culex pipiens*-*Plasmodium relictum*) and wild (mosquitoes hatched from clutches collected on the field and *Plasmodium* strain was isolated since two years) vector-parasite association. The use of a natural vertebrate host (instead of *Serinus canaria*, an artificial host used here for practical reasons) would allow to study a fully natural host-vector-parasite association. As already stressed in the literature [5], there are important factors to consider that may explain the existence of conflicting results between studies.

## Conclusions

In this study, we showed a dual response after the ingestion of a *Plasmodium*-infected blood meal in the mosquito vector. Following exposure, an allocation trade-off occurred between self-maintenance and reproduction, which tended to lead either to investing in defences but not reproducing or to reproducing more efficiently under infection risk. We also showed an involvement of immune and/or antioxidant defences that may act as mediators. These results illustrate a complex effect of exposure to *Plasmodium* on vector life history traits. The understanding of the mechanisms underlying the variation between individuals, but also between studies, is particularly important for malaria epidemiology.

## Additional files

Additional file 1: Figure S1. Bird parasitaemia and haematocrit. **a** Parasitaemia (log-transformed, arbitrary unit) before the first feeding session, at the end of all the feeding sessions and mean value for canaries in the chronic group (1 to 4) and in the acute group (5 to 8). Mean values significantly differed between the two groups (Wilcoxon rank test: *W* = 0, *P* = 0.029). **b** Haematocrit (fraction of red blood cells in the total blood volume) before the first feeding session, at the end of all the feeding sessions and mean value for canaries in the chronic group (1 to 4), in the acute group (5 to 8) and in the uninfected group (9 to 12). Mean values did not significantly differ between the three groups (*F*_(2,9)_ = 1.08, *P* = 0.381). (PDF 183 kb)
Additional file 2: Table S1. Detailed model structures and initial full models prior to model selection. For each response variable (blood meal size, clutch size, survival and laying probability), details are given about which females (♀) have been considered, the model type as well as the fixed and the random factors considered. **a** for each response variable, the same models were also ran considering females under low and high exposure only, replacing exposure level by bird parasitaemia and removing canary identity from the random factors. **b** *Abbreviations*: *dpf*: days post-feeding, *NOS*: nitric oxide synthase, *SOD*: superoxide dismutase, *G6PDH*: glucose-6-phosphate dehydrogenase. (DOCX 13 kb)

## Abbreviations

cDNA: Complementary deoxyribonucleic acid
DNA: Deoxyribonucleic acid
dpf: Days post-feeding
G6PDH: Glucose-6-phosphate dehydrogenase
NADP: Nicotinamide adénine dinucléotide phosphate
NO: Nitric oxide
NOS: Nitric oxide synthase
NOX: NADPH-oxidase
O_2_^−^: Superoxide
PBS: Phosphate buffer saline
PCR: Polymerase chain reaction
qPCR: Quantitative polymerase chain reaction
RNA: Ribonucleic acid
SOD: Superoxide dismutase
