# Supplementary figures and images for: Exposure of the mosquito vector Culex pipiens to the malaria parasite Plasmodium relictum: effect of infected blood intake on immune and antioxidant defences, fecundity and survival
Source: Parasit Vectors. 2016 Nov 29;9:616. doi: 10.1186/s13071-016-1905-7 (PMC5129600; doi:10.1186/s13071-016-1905-7)

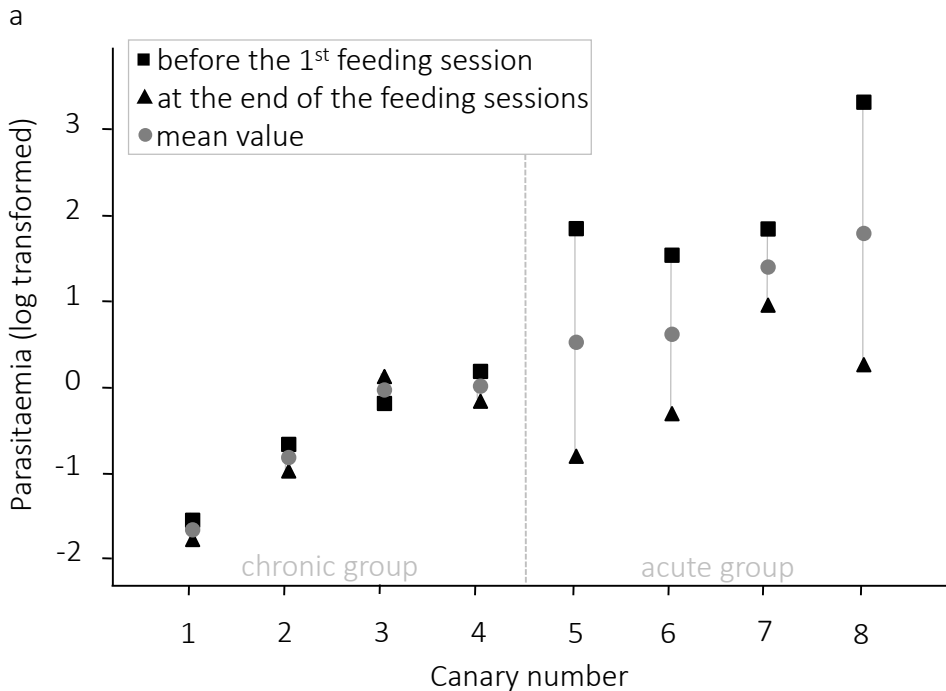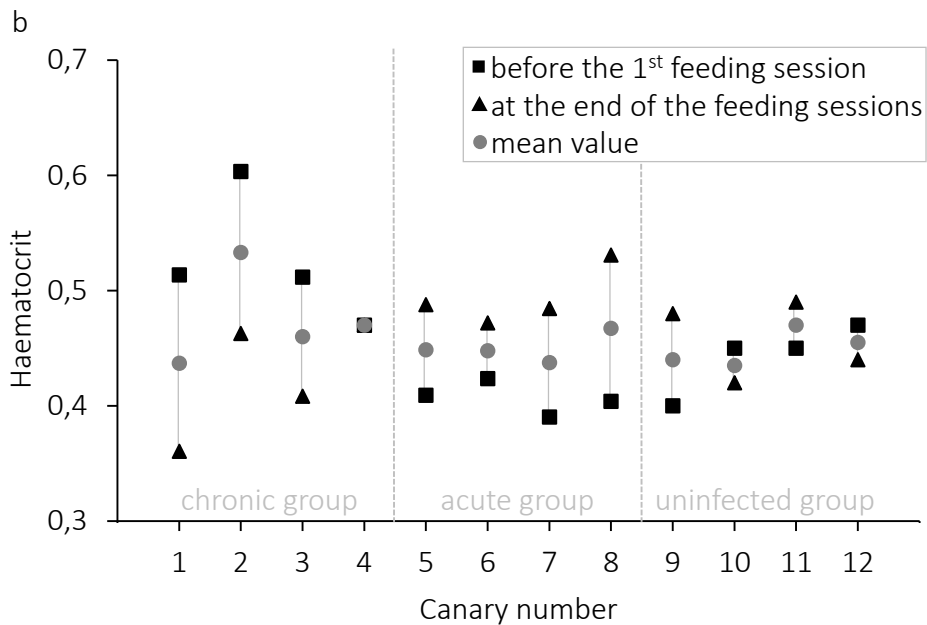

Supplement: Additional file 1: Figure S1. — Bird parasitaemia and haematocrit. a Parasitaemia (log-transformed, arbitrary unit) before the first feeding session, at the end of all the feeding sessions and mean value for canaries in the chronic group (1 to 4) and in the acute group (5 to 8). Mean values significantly differed between the two groups (Wilcoxon rank test: W = 0, P = 0.029). b Haematocrit (fraction of red blood cells in the total blood volume) before the first feeding session, at the end of all the feeding sessions and mean value for canaries in the chronic group (1 to 4), in the acute group (5 to 8) and in the uninfected group (9 to 12). Mean values did not significantly differ between the three groups (F (2,9) = 1.08, P = 0.381). (PDF 183 kb) [file 13071_2016_1905_MOESM1_ESM.pdf]
