# Supplementary material for: Exposure of the mosquito vector Culex pipiens to the malaria parasite Plasmodium relictum: effect of infected blood intake on immune and antioxidant defences, fecundity and survival
Source: Parasit Vectors. 2016 Nov 29;9:616. doi: 10.1186/s13071-016-1905-7 (PMC5129600; doi:10.1186/s13071-016-1905-7)
Supplement: Additional file 2: Table S1. — Detailed model structures and initial full models prior to model selection. For each response variable (blood meal size, clutch size, survival and laying probability), details are given about which females (♀) have been considered, the model type as well as the fixed and the random factors considered. a for each response variable, the same models were also ran considering females under low and high exposure only, replacing exposure level by bird parasitaemia and removing canary identity from the random factors. b Abbreviations: dpf: days post-feeding, NOS: nitric oxide synthase, SOD: superoxide dismutase, G6PDH: glucose-6-phosphate dehydrogenase. (DOCX 13 kb) [file 13071_2016_1905_MOESM2_ESM.docx]

**Additional file 2. Table S1.**

| Response variables | | | Fixed factors | Random factors |
| --- | --- | --- | --- | --- |
| a | | |  |  |
|  | Blood meal size (square root transformed) | |  |  |
|  | Considering | All fed ♀ | Body size | Emergent group |
|  | Model | lmer | Exposure level | Canary identity |
|  |  |  | Body size : exposure |  |
|  | Clutch size | |  |  |
|  | Considering | ♀ which oviposited | Body size | Emergent group |
|  | Model | lmer | Blood meal size | Canary identity |
|  |  |  | Exposure level |  |
|  |  |  | Body size : exposure |  |
|  |  |  | Blood meal size : exposure |  |
|  | Survival | |  |  |
|  | Considering | ♀ which died naturally | Blood meal size | Emergent group |
|  | Model | coxme | Clutch size | Canary identity |
|  |  |  | Exposure level |  |
|  |  |  | Blood meal size : exposure |  |
|  |  |  | Clutch size : exposure |  |
|  | Laying probability | |  |  |
|  | Considering | ♀ which died naturally | Blood meal size | Emergent group |
|  | Model | glmer | Exposure level | Canary identity |
|  |  |  | Blood meal : exposure |  |
| b | | |  |  |
|  | Clutch size | |  |  |
|  | Considering | Exposed ♀  sacrificed at 15 dpf  which oviposited | NOS level  SOD level  G6PDH level | Emergent group  Canary identity |
|  | Model | lmer | Exposure level |  |
|  |  |  | NOS : exposure |  |
|  |  |  | SOD : exposure |  |
|  |  |  | G6PDH : exposure |  |
|  | Laying probability | |  |  |
|  | Considering | Exposed ♀  sacrificed at 15 dpf | NOS level  SOD level | Emergent group  Canary identity |
|  | Model | glmer | G6PDH level |  |
|  |  |  | Exposure level |  |
|  |  |  | NOS : exposure |  |
|  |  |  | SOD : exposure |  |
|  |  |  | G6PDH : exposure |  |
